# The role of circular RNAs in regulating resistance to cancer immunotherapy: mechanisms and implications

**DOI:** 10.1038/s41419-024-06698-3

**Published:** 2024-05-02

**Authors:** Yu Ma, Ting Wang, Xudong Zhang, Pinghan Wang, Fangyi Long

**Affiliations:** 1 Department of Clinical Research, Sichuan Clinical Research Center for Cancer, Sichuan Cancer Hospital & Institute, Sichuan Cancer Center, Affiliated Cancer Hospital of University of Electronic Science and Technology of China, Chengdu, 610041 China; 2 Laboratory Medicine Center, Sichuan Provincial Maternity and Child Health Care Hospital, Affiliated Women’s and Children’s Hospital of Chengdu Medical College, Chengdu Medical College, Chengdu, 610032 China

**Keywords:** Cancer therapeutic resistance, miRNAs

## Abstract

Cancer immunotherapy has rapidly transformed cancer treatment, yet resistance remains a significant hurdle, limiting its efficacy in many patients. Circular RNAs (circRNAs), a novel class of non-coding RNAs, have emerged as pivotal regulators of gene expression and cellular processes. Increasing evidence indicates their involvement in modulating resistance to cancer immunotherapy. Notably, certain circRNAs function as miRNA sponges or interact with proteins, influencing the expression of immune-related genes, including crucial immune checkpoint molecules. This, in turn, shapes the tumor microenvironment and significantly impacts the response to immunotherapy. In this comprehensive review, we explore the evolving role of circRNAs in orchestrating resistance to cancer immunotherapy, with a specific focus on their mechanisms in influencing immune checkpoint gene expression. Additionally, we underscore the potential of circRNAs as promising therapeutic targets to augment the effectiveness of cancer immunotherapy. Understanding the role of circRNAs in cancer immunotherapy resistance could contribute to the development of new therapeutic strategies to overcome resistance and improve patient outcomes.

## Facts

- CircRNAs play a significant role in modulating immune-related pathways and the tumor microenvironment in cancer immunotherapy resistance.
- Dysregulation of circRNAs can profoundly impact tumor progression, making therapeutic strategies targeting circRNA highly promising for clinical applications.
- CircRNA research is still in its early stages, and clinical applications, particularly in terms of personalized treatment, are yet to be fully realized.

## Open questions

- What are the precise molecular mechanisms through which circRNAs modulate immune checkpoints and tumor microenvironment factors in different cancer types?
- What are the implications of circRNA dysregulation in specific pathological stages of cancer progression for immunotherapy efficacy?
- How can advanced methods for the design, synthesis, purification, and delivery of circRNA-based therapies be developed to enhance their clinical translation?

## Introduction

Cancer is a significant global health challenge, annually claiming millions of lives [1]. The emergence of immunotherapy has ushered in a transformative era in oncology treatment, complementing traditional methods like surgery, radiotherapy, chemotherapy, and targeted therapy. This paradigm shift leverages the innate capability of the immune system to identify and eradicate cancer cells, offering a ray of hope [2]. However, despite notable successes in treating various cancers types, such as lung cancer, melanoma, and hepatocellular carcinoma, resistance remains a significant challenge [3]. As the most promising immunotherapy, the efficacy of anti-programmed death-1/programmed death ligand 1 (anti-PD-1/L1) therapies is confined to a minority of patients, with sustained positive responses observed in only 10–30% of cases [4]. Thus, a comprehensive understanding of the intricate mechanisms underlying immunotherapy resistance is imperative for devising effective treatment strategies.

Recent studies have spotlighted the pivotal role of circular RNAs (circRNAs) in orchestrating resistance to cancer immunotherapy [5]. CircRNAs, a unique class of endogenous non-coding RNAs forming covalently closed circular structures, defy the linear structure of other RNA molecules [6]. Initially deemed splicing artifacts, recent studies have unveiled their critical role in regulating gene expression by acting as miRNA sponges, RNA-binding protein sequestering agents, or transcriptional regulators. Dysregulation of circRNAs has been noted in various cancer types, with several studies illustrating their involvement in cancer progression and metastasis [7, 8]. Emerging evidence suggests that circRNAs also play a crucial role in regulating resistance to cancer immunotherapy. They can modulate various aspects of tumor-immune interactions, including immune cell infiltration, immune checkpoint regulation, and cytokine signaling pathways, thereby influencing the response to immunotherapy.

In this review, we provide an overview of the emerging role of circRNAs in regulating resistance to cancer immunotherapy. We discuss the current understanding of the mechanisms by which circRNAs modulate tumor-immune interactions and the impact of these interactions on the response to immunotherapy. Additionally, we highlight the potential of circRNAs as biomarkers for predicting response to immunotherapy and as therapeutic targets for overcoming resistance. Overall, this review offers a comprehensive understanding of the role of circRNAs in regulating resistance to cancer immunotherapy and their potential as therapeutic targets and biomarkers.

## CircRNAs: biogenesis and functions (Fig. 1)

### Overview of circRNA biogenesis and features

CircRNAs constitute a subtype of non-coding RNA characterized by a covalently closed-loop structure. In 1976, Sanger et al. first identified circular RNA molecules in nature through electron microscopy [9]. Subsequent decades revealed the presence of circRNAs in yeast mitochondria [10], Hepatitis delta virus [11], and genes capable of producing circRNA, such as the human dystrophin gene [12] and the mouse sex-determining region Y (Sry gene) [13]. Formed through back-splicing events, circRNAs involve the joining of the 3’ end of a downstream exon with the 5’ end of an upstream exon, resulting in a circular molecule [14, 15]. Initially perceived as rare splicing by-products, recent studies demonstrate their abundance and widespread expression across various tissues and organisms [16].

**Fig. 1:**
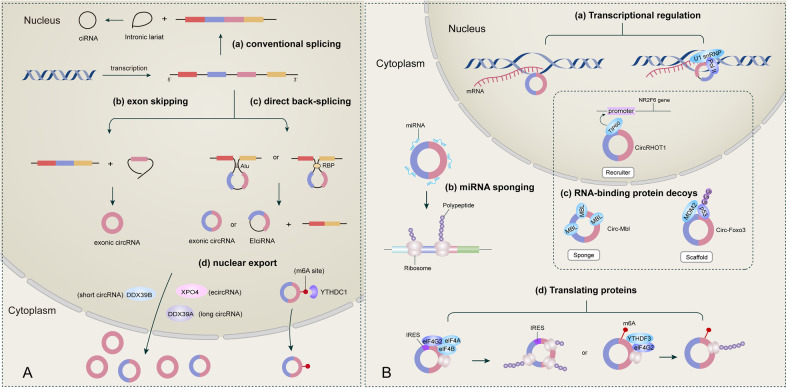
Biogenesis and function of circRNAs. **A** Biogenesis of circRNAs. (a) conventional splicing. The conventional splicing process involves the formation of intronic lariats, escaping debranching and ligation, resulting in the creation of intronic circRNAs (CiRNAs). (b) exon skipping. (c) direct back-splicing. (d) nuclear export. The nuclear export of short (<400-nt) and long (>1200-nt) circRNAs is mediated by DDX39A and DDX39B, respectively. Another crucial regulator of nuclear export is the conserved Exportin 4 (XPO4). Additionally, m6A modification can facilitate the nuclear export of circRNAs. **B** Functions of circRNAs. (a) Transcriptional regulation: circRNAs can influence parental gene transcription by forming a three-stranded R-loop structure with its production site or by interacting with transcriptional complexes, such as U1 snRNP. (b) miRNA sponges: by competitively binding to miRNAs, circRNAs up-regulate downstream target mRNA and corresponding proteins, ultimately impacting cellular physiological processes. (c) RNA-binding protein decoys: acting as sponges, scaffolds, and recruiters, circRNAs interact with RNA-binding proteins, regulating various intracellular physiological processes. (d) Translating proteins: circRNAs exhibit two cap-independent translation modes: IRES-mediated and m6A-mediated. In the IRES-mediated process, IRES binds to the initiation factor eIF4G2, assembles with eIF4A and eIF4B, recruits the 40 s ribosome subunit, and forms a 43 s initiation complex that initiates translation upon encountering the initiation codon ATG. Another translation type is mediated by the m6A motif in circRNA, where m6A is recognized by the m6A reader YTH domain family protein 3 (YTHDF3), recruiting initiation factors and ribosome subunits to form translation-initiation complexes inducing translation.

The biogenesis of circRNAs relies on non-canonical spliceosome machinery, regulated by trans-acting proteins and cis-regulatory elements. The interplay between back splicing and canonical splicing, mediated by splicing factors, determines the equilibrium between the two processes [17]. Based on the sequence order of back splicing and canonical splicing, circRNA biosynthesis is categorized into two models: (1) Lariat-driven circularization (or the exon-skipping model) and direct back-splicing. In the Lariat-driven circularization model, canonical splicing precedes, producing a linear RNA with skipped exons and a lariat precursor. This precursor, containing introns and exons, undergoes back-splicing to generate a circRNA. (2) In the direct back-splicing model, back splicing occurs first, directly producing a circRNA and a linear exon-intron(s)-exon intermediate. This intermediate can undergo further splicing, resulting in a linear RNA with skipped exons [5, 18, 19].

CircRNAs are categorized into three main types based on their origin: exonic circRNAs (ecircRNAs), circular intronic RNAs (ciRNAs), and exon-intron circRNAs (EIciRNAs). EcircRNAs comprise one or more exons and are primarily located in the cytoplasm, while ciRNAs and EIciRNAs are situated in the nucleus. EcircRNAs predominate among the total circRNAs [20–22]. CircRNAs exhibit distinctive features, such as a covalently closed-loop structure conferring stability and resistance to Rnase degradation [23]. The longer half-lives of circRNAs (18.8–23.7 h) compared to their linear counterparts (4.0–7.4 h) contribute to their enhanced stability [24]. Additionally, circRNAs display tissue-specific and developmental stage-specific expression, with high levels in specific cell types, such as nerve cells in the brain. Many circRNAs are evolutionarily conserved, underscoring their potential functional roles [25, 26]. Furthermore, circRNAs vary in length, originate from different exons or introns, and exhibit diverse sequences, contributing to a wide range of potential functions. Serving as miRNA sponges, RNA-binding protein (RBP) decoys, or splicing modulators, circRNAs emerge as a diverse class of non-coding RNAs with crucial regulatory roles in eukaryotic gene expression.

### Functions of circRNAs

CircRNAs have emerged as pivotal regulators of gene expression in eukaryotes, demonstrating diverse functions across various aspects:

MiRNA sponges: circRNAs function as competing endogenous RNAs (ceRNAs) that sequester miRNAs, thereby preventing them from targeting their mRNA counterparts. This sequestration leads to increased expression of mRNA targets, impacting cellular processes such as proliferation, differentiation, and apoptosis. MiRNA sponge circRNAs, usually exhibiting high expression, contain numerous microRNA response elements (MREs) [5, 6, 20]. A classic example is ciRS-7, which harbors over 70 selectively conserved miRNA target sites, inhibiting miR-7 activity and resulting in elevated levels of miR-7 targets [27]. Studies indicate that circRNAs act as miRNA sponges to either inhibit or promote tumor growth. For example, circCD44 directly binds to miR-502-5p, promoting the proliferation, migration, and invasion of triple-negative breast cancer [28]. Another instance is circMTO1, which inhibits liver cancer by sponging miR-9, subsequently up-regulating the expression of the tumor suppressor p21 [29]. Growing evidence supports the notion that circRNA-miRNA interactions are a universal regulatory mechanism.

RNA-binding protein decoys: circRNAs can act as decoys for RNA-binding proteins (RBPs), preventing their binding to target mRNAs. This interaction alters the stability or translation of mRNA targets, influencing cellular processes. CircRNAs interact with RBPs as protein sponges, scaffolds, and recruiters [6]. The initial example of circRNAs acting as protein sponges is circMbl, where the splicing factor muscleblind (MBL) and its flanking introns possess conserved MBL binding sites. Elevated MBL concentrations promote circMbl generation while reducing linear mRNA MBL production. Highly expressed circMbl also adsorbs MBL, impeding its neural function [6, 17, 20]. As protein scaffolds, circRNAs facilitate interactions between different proteins. For instance, circ-Foxo3 in breast cancer cells binds to p53 and E3 ubiquitin ligase mouse doubleminute 2 homolog (MDM2), forming a ternary complex and promoting p53 ubiquitination and degradation by MDM2 [30]. Additionally, circRNAs can recruit specific proteins to particular sites. For example, circRHOT1 induces liver cancer development by recruiting TIP60 (also known as histone acetyltransferase KAT5) to the promoter of nuclear receptor subfamily 2 group F member 6 (NR2F6), thereby inducing NR2F6 expression [6, 31]. In summary, circRNA-protein interactions can influence target gene expression, thereby affecting human disease development.

Transcriptional regulation: circRNAs can regulate gene expression by interacting with transcription factors or chromatin modifiers to impact target gene transcription. On one hand, circRNAs can influence transcription by forming a three-stranded R-loop structure with their production site. For instance, Ci-ankrd52 binds to the parental locus ANKRD52, forming an R-loop, activating RNase H1-mediated ci-ankrd52 digestion, disrupting the R-loop, and promoting transcriptional extension [32]. On the other hand, circRNAs can activate transcription factors (TFs). For instance, circPOK (encoded by the Zbtb7a gene) co-activates the ILF2/3 complex, binding to the proximal promoter of II6 [33]. Additionally, EIciRNAs enhance the transcription of parental genes by interacting with U1 small nuclear ribonucleoprotein (U1 snRNP), such as circEIF3J and circPAIP21922. Knocking down circEIF3J and circPAIP2 decreases the transcription levels of EIF3J and PAIP2, respectively [34].

Translating proteins: while circRNAs are generally considered non-coding due to the lack of a 5’ cap and 3’ poly(A) tail [35], existing studies suggest that circRNAs can be translated through cap-independent mechanisms mediated by internal ribosome entry sites (IRES) [36], as well as by N6-methyladenosine (m6A) modification [37]. In the IRES-mediated process, IRES binds to the initiation factor eIF4G2, assembling with eIF4A and eIF4B to recruit 40 s ribosomal subunits and form the 43 s initiation complex, initiating translation upon encountering the initiation codon ATG [38]. Another non-cap translation type is predominantly mediated by m6A located in the 5’-untranslated region. This m6A is recognized by the m6A reader YTH domain family protein 3, recruiting initiation factors and ribosomal subunits to form translation initiation complexes, inducing translation [39]. CircRNAs-mediated protein translation can regulate tumor growth. For instance, the study by Yang Y et al. demonstrates that circFBXW7 encodes the functional protein FBXW7-185aa, which competes with FBXW7α for deubiquitinase USP28, resulting in free FBXW7α-induced ubiquitination degradation of c-Myc and inhibiting glioblastoma development in the brain [40]. It is noteworthy that circRNAs translate proteins to maintain cell survival under stress conditions such as hypoxia, heat shock, or viral infection [41]. However, under non-stress conditions, circRNAs may not undergo translation due to the predominance of cap-dependent translation [42].

Based on these functions, circRNAs are implicated in various diseases, including cancer, cardiovascular disease, and neurological disorders. Recently, circRNAs have emerged as potential targets for cancer therapy, given their involvement in cellular processes related to cancer progression, such as cell proliferation, invasion, metastasis, and drug resistance. For instance, circHIPK3 is upregulated in various cancer types and promotes resistance to chemotherapy and radiotherapy by sponging multiple miRNAs [43, 44]. Targeting this circRNA could sensitize cancer cells to these treatments. CircVAPA is upregulated in non-small cell lung cancer, acting as a sponge for miR-377-3p and miR-494-3p, thereby accelerating tumor proliferation and differentiation through the IGF1R/AKT axis. Knockdown of circVAPA significantly enhances the effect of IGF1R kinase inhibitor (BMS-536924) in inhibiting tumor growth [45]. These studies underscore the potential of targeting circRNAs for cancer therapy. Nevertheless, further research is needed to comprehensively understand circRNA regulation mechanisms in cancer and develop effective circRNA-targeting therapies.

## Cancer immunotherapy resistance

### Cancer immunotherapy (Fig. 2)

The immune system plays a critical role in protecting the body against cancer. T-cells are one of the key components of the immune system, as they are responsible for recognizing and attacking abnormal or infected cells, including cancer cells. However, cancer cells can often evade detection by the immune system through two main mechanisms: either by producing signals that suppress the immune response or by presenting themselves as “self” to the immune system to avoid being recognized as foreign. Cancer immunotherapy utilizes the body’s immune system to fight cancer. It works by stimulating the immune system to recognize and attack cancer cells. There are several different types of cancer immunotherapy, including immune checkpoint inhibitors (ICIs), adoptive cell therapy (ACT), cancer vaccines, oncolytic virus therapy, and immune system modulators.

**Fig. 2:**
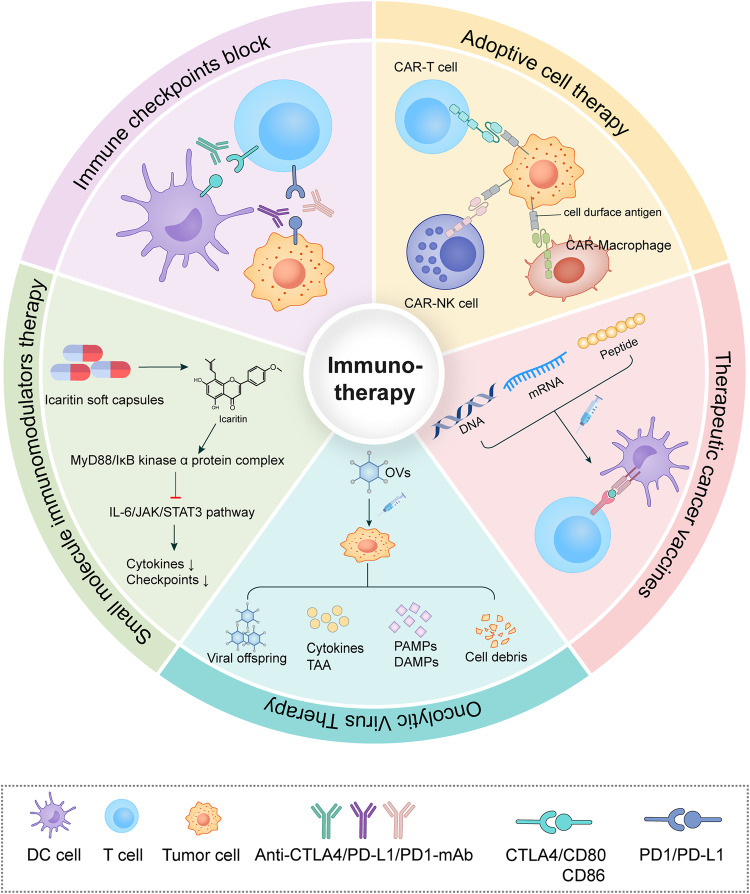
Cancer immunotherapy. Immune checkpoint blockade. Immune checkpoint inhibitors bind to immunosuppressive proteins on the cell surface, restoring the antitumor function of T cells. Adoptive cell therapy: CAR-immune cells are engineered to specifically target antigens on the surface of tumor cells, enhancing the immune system’s ability to combat cancer. Therapeutic cancer vaccines: Therapeutic cancer vaccines deliver antigens to Antigen-Presenting Cells (APCs), activating and inducing cytotoxic T lymphocyte responses to fight against cancer cells. Oncolytic virus therapy: Oncolytic viruses cause oncolysis, releasing viral offspring, Pathogen-Associated Molecular Patterns (PAMPs), Damage-Associated Molecular Patterns (DAMPs), and Tumor-Associated Antigens (TAAs). This stimulates the immune system to target and destroy cancer cells. Small molecule immunomodulators: Illustrated by icaritin soft capsules, icaritin targets the MyD88/IL-6/JAK/STAT3 signaling pathway. This results in reduced cytokine production (e.g., TNF-α and IL-6) and a decrease in the expression of immune checkpoints (e.g., PD-L1), contributing to enhanced antitumor immunity.

#### Immune checkpoints block (ICB) therapy

ICIs can block specific proteins on immune cells that prevent them from attacking cancer cells. Immune checkpoints are cell surface receptors expressed by immune cells, a group of co-stimulatory signals, including stimulatory and inhibitory molecules, that regulate the activation and effector functions of immune cells, such as cytotoxic T lymphocyte-associated antigen-4 (CTLA-4), programmed death-1/programmed death ligand 1 (PD-1/PD-L1), and T cell immunoglobulin and mucin-3 (Tim-3) [46]. Under normal circumstances, immune checkpoints (ICPs) maintain self-tolerance and immune homeostasis, but when malignant tumors occur, ICPs are occupied, such as PD-L1 on tumor cells binding to PD-1 on T cells and thus preventing effector T cells from functioning, allowing tumor cells to achieve immune escape and promoting tumor growth [47]. Blockade of immune checkpoints can reactivate tumor immunity. Currently, the main ICIs commonly used in clinical practice target CTLA4, PD-1 and PD-L1, such as ipilimumab, nivolumab, and durvalumab [48]. For the remarkable results of ICIs in anti-tumor, James P. Allison and Tasuku Honjo, two scientists working on CTLA-4 and PD-1, respectively, were also awarded the 2018 Nobel Prize in Medicine and Physiology [49]. At present, ICIs are also approved for a growing number of indications, including metastatic melanoma, renal cell carcinoma, advanced non-small cell lung cancer, bladder cancer, lymphoma, and more [50]. The simultaneous use of different types of ICIs can also exert synergistic anti-tumor effects. For example, anti-PD-1/L1 therapy combined with anti-CTLA4 has become the standard for the treatment of melanoma, and the combination of nivolumab and ipilimumab in patients with advanced melanoma can improve the survival rate of patients of 5 years [51].

#### Adoptive cell therapy (ACT)

ACT involves removing immune cells from a patient, growing them in a lab, and then infusing them back into the patient to attack cancer cells [52]. ACT includes tumor infiltrating lymphocytes (TILs) therapy, endogenous T cells therapy, T cell receptor therapy, and chimeric antigen receptor (CAR) T cell therapy [53]. One of the most promising therapeutic methods is CAR-T therapy, which genetically modifies a patient’s T cells to target specific antigens on tumor cells. CAR-T cells do not rely on major histocompatibility complex (MHC) molecules to recognize antigens, which overcomes the problem of missing the expression of MHC class I molecules in tumor cells [54]. This method has shown good efficacy in patients with chemotherapy-refractory tumors of hematologic malignancies such as leukemia, myeloma, and non-Hodgkin B-cell lymphoma [55]. At present, in addition to CAR-T, CAR-natural killer cells (CAR-NK), and CAR-macrophages (CAR-M) have been introduced as a supplement or alternative to CAR-T cell therapy [52].

#### Therapeutic cancer vaccines

Therapeutic tumor vaccines are a safe way to boost T-cell responses and produce therapeutic effects at all stages of disease in tumor patients [56]. Tumor vaccines consist of tumor antigen, formulation, immune adjuvant, and delivery vehicle [57]. There are four main vaccine types: tumor whole-cell vaccines, genetically engineered vaccines, protein peptide vaccines, and dendritic cell vaccines [58]. Successful therapeutic cancer vaccines require high-quality antigens to be delivered to antigen-presenting cells and activated and induce cytotoxic T lymphocyte responses, maintain immune cell infiltration in tumor microenvironment (TME), and ultimately induce tumor regression, build durable anti-tumor memories, and avoid non-specific or adverse reactions [59]. Ideally, vaccines would target tumor-specific antigens to avoid an autoimmune response, but many vaccines target tumor-associated antigens (TAAs), which are often recognized by the immune system as ‘self’ [56]. Although flawed, this does not negate the therapeutic effectiveness of tumor vaccines. In a Phase I dose-escalation trial (NCT02410733), the mRNA lipid complex vaccine (BNT111) exhibited promising safety and efficacy in advanced melanoma patients, with an immune response against one or more tumor-associated antigens detected in over 75% of participants. Although therapeutic mRNA vaccines are not yet standard, their combination with ICIs in clinical trials has shown satisfactory results [60].

#### Oncolytic virus therapy

Oncolytic virus (OVs) immunotherapy exploits viruses to selectively infect and replicate within tumor cells, leading to tumor cell lysis, the release of tumor antigens, and the activation of the immune system through additional damage-associated molecular patterns and viral pathogen-associated molecular patterns (PAMPs) [61]. OVs, either naturally occurring or genetically modified, selectively eliminate tumor cells and induce host anti-tumor immunity [62]. Common OVs include newcastle disease viruses, herpes viruses, coxsackievirus, measles viruses, adenoviruses, polioviruses, poxviruses, and reoviruses [63]. Genetically engineered OVs enhance tumor selectivity, promote replication ability, and increase immunogenicity [64]. In clinical settings, OVs are often combined with other treatment methods to enhance treatment sensitivity. For instance, in a phase II clinical trial, the combination of talimogene laherparepvec and ipilimumab outperformed ipilimumab alone, increasing the objective response rate from 18 to 39% in patients with advanced melanoma [65].

#### Small molecule immunomodulators therapy

Most current immunotherapies rely on antibodies, which, while exhibiting specificity and efficacy in pharmacodynamics, face limitations in pharmacokinetics, such as extended half-life and poor tumor tissue permeability. To overcome these challenges, researchers have developed small molecule-based immunotherapy methods. The combined use of small molecule immunomodulators and antibody drugs produces synergistic effects, representing a complementary mode of tumor therapy [66]. The therapeutic strategy of small molecule immunomodulators involves targeting intracellular and extracellular molecules to impact various intracellular signaling pathways, thereby inhibiting tumor growth. Examples include small molecule PD-L1 inhibitors, PD-L1-targeting PROTAC degraders, chemokine receptor antagonists, RORγt agonists, small molecule TGF-β inhibitors, small molecule STING agonists, etc. Although many of these are in clinical trials, no small molecule-based cancer immunotherapies have yet gained approval from the US Food and Drug Administration (FDA) [66]. However, a Chinese herbal monomer preparation named icaritin soft capsules has been approved as an immunomodulatory agent for treating advanced hepatocellular carcinoma (HCC) in China [67]. Icaritin interacts with the MyD88/IкB kinase α protein complex, suppresses the IL-6/JAK/STAT3 signaling pathway, reduces cytokine generation, and decreases the expression of immune checkpoints. Furthermore, icaritin inhibits the bioactivity of myeloid-derived suppressor cells (MDSCs) by down-regulating tumor-associated splenic extramedullary hematopoiesis [67].

In instances where a single immunotherapy approach proves less effective, a combination of therapies, such as combining immunotherapy with targeted therapy, radiotherapy, or chemotherapy, can be chosen. Detailed combination strategies that maximize the benefits of immunotherapy are reviewed by Zhu et al. and Yap et al. [68, 69]. Ongoing research is directed toward enhancing the effectiveness and safety of immunotherapy and identifying patients most likely to benefit from this form of treatment [70].

### Mechanisms underlying resistance to cancer immunotherapy (Fig. 3)

Resistance to cancer immunotherapy refers to the failure of a patient’s immune system to effectively target and eliminate cancer cells, despite treatment with immunotherapeutic agents. The mechanisms underlying resistance are complex and multifaceted, stemming from various factors. Here, we will introduce several resistance mechanisms prevalent in various types of tumor immunotherapy.

**Fig. 3:**
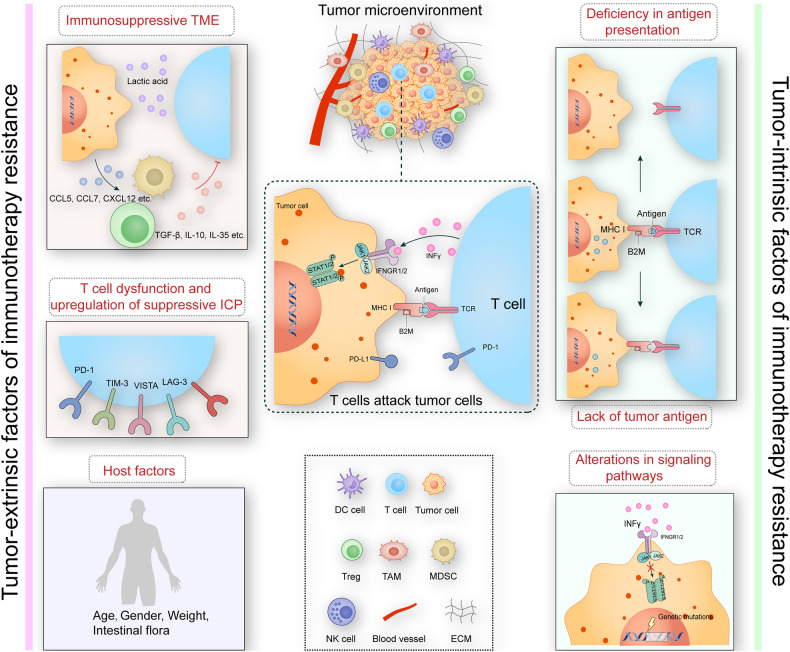
Mechanisms of resistance to tumor immunotherapy. T cells, pivotal in recognizing and attacking tumor cells through TCR-mediated MHC-binding peptide antigens (center), encounter resistance mechanisms originating from intrinsic (right) and extrinsic factors (left). Internal factors, primarily arising from defective antigen presentation, antigen deletion, and signaling pathway alterations due to gene mutations, collectively result in compromised T-cell recognition and response against tumor cells. External factors encompass T-cell dysfunction, up-regulation of inhibitory immune checkpoints, and the impact of the immunosuppressive Tumor Microenvironment (TME) and host factors. T-cell dysfunction and heightened inhibitory immune checkpoints contribute to immune escape by tumor cells. The immunosuppressive TME, characterized by nutrient deficiency, hypoxia, acidity, and a plethora of immunosuppressive cells, fosters an environment detrimental to antitumor immune responses. The acidic microenvironment, emanating from lactic acid release by tumor cells, inhibits the cytotoxicity and proliferation of CD8+ T cells. Moreover, lactate and chemokines (e.g., CCL5, CCL7, CXCL12) secreted by tumor cells orchestrate the recruitment and induction of immunosuppressive cells like Treg cells, tumor-associated macrophages, and myeloid-derived suppressor cells into the TME. These recruited cells further secrete inhibitory cytokines (e.g., TGF-β, IL-10, IL-35), hindering the functionality of T cells. Host factors predominantly involve patient-specific elements such as gender, age, weight, and gastrointestinal flora, all contributing to the complex landscape of tumor immunotherapy resistance.

#### Tumor-intrinsic factors of immunotherapy resistance

##### Lack of tumor antigen and reduced immunogenicity

tumor immunogenicity, correlated with T cell recognition, hinges on the tumor’s ability to produce neoantigens—an integral factor determining immunotherapy response [71]. The loss of tumor antigens significantly impedes T-cell recognition and functionality, resulting in immunotherapy failure [72]. High immunogenic tumors, such as melanoma and kidney cancer, exhibit better immunotherapy efficacy, while tumors with low immunogenicity, like pancreatic and prostate cancer, are less responsive to ICI treatment [73]. Microsatellite instability (MSI) in tumors with mismatch repair deficiency (MMR) can lead to high tumor mutational burden (TMB), enhancing immunogenicity and ICI therapy response [72, 74]. MSI and TMB serve as biomarkers predicting immunotherapy efficacy [75].

##### Deficiency in antigen presentation

the antigen peptide-MHC I complex activation of CD8+ T cells is crucial for anti-tumor efficacy [76]. Mutations in genes involved in antigen processing and presentation, including MHC molecules, beta 2 microglobulin (B2M), large multifunctional protease (LMP), and transporter associated with antigen processing (TAP), contribute to ICI resistance [73]. Notably, B2M gene mutations, particularly homozygous truncation, impede MHC Class I molecule expression, affecting antigen presentation and ICI response [77]. Downregulation or loss of B2M expression has been observed in resistant cases, impacting MHC I or HLA I expression [78, 79]. For instance, CD19 deletion in B-cell acute lymphoblastic leukemia renders CART19 treatment ineffective [80].

##### Alterations in signaling pathways

various signaling pathway alterations, such as enhanced PI3K/AKT signaling and loss of interferon-gamma (IFN-γ) signaling, contribute to immunotherapy resistance. PTEN loss in melanoma activates PI3K/AKT, suppressing T cell infiltration and autophagy, leading to resistance [81]. IFN-γ, vital for anti-tumor effects, loses effectiveness when tumors acquire JAK1/2 loss-of-function mutations, hindering IFN-γ-induced PD-L1 expression and MHC-I molecules [79, 82, 83]. WNT/β-catenin and MAPK signaling also associate with immunotherapy resistance [84].

#### Tumor-extrinsic factors of immunotherapy resistance: tumor microenvironment

##### T cell dysfunction and upregulation of suppressive immune checkpoint expression

Prolonged antigenic stimulation induces abnormal T cell function, marked by reduced proliferative capacity, diminished effector function, and increased inhibitory receptor expression [85]. Disruptions in T cell immune function, including antigen recognition, activation, differentiation, and chemotaxis, may result in ineffectiveness of anti-PD therapy [86]. Studies have also shown that blockade of a single ICP leads to compensatory upregulation of other ICPs and affects the efficacy of drug therapy [87]. For example, in the anti-PD-1 treatment of head and neck tumors, TIL compensatoryly upregulates Tim-3 in a PI3K/Akt-dependent manner. Tim-3 inhibits T cell activation by inhibiting the phosphorylation of Akt/S6, thereby making anti-PD-1 therapy resistant [88]. It has been reported that the combination of anti-PD-1 and anti-Tim-3 will produce better therapeutic effects [89]. Upregulation of other immune checkpoints, PD-1/PD-L1, VISTA, TIGIT, LAG-3, also promotes immunotherapy resistance [90]. For example, upregulated expression of VISTA is one of the factors responsible for resistance to anti-PD-1 therapy in patients with metastatic melanoma[91].

##### Immunosuppressive tumor microenvironment

The TME, comprising tumor and stromal cells, immune cells, blood vessels, and signaling molecules, plays a pivotal role in immunotherapy resistance. An immunosuppressive TME, marked by nutritional deficiencies, hypoxia, acidity, and abundant immunosuppressive cells (Treg cells, tumor-associated macrophages, myeloid-derived suppressor cells), impedes anti-tumor responses and promotes resistance [92–94]. Tumor cells reshape the TME through metabolic processes like aerobic glycolysis (Warburg effect), creating an acidic environment that hampers CD8+ T cell cytotoxicity and proliferation, inhibiting immunotherapy sensitivity [95]. Tumor cells also influence the TME by secreting chemokines that recruit immunosuppressive cells, affecting effector T cell function. For instance, MDSCs inhibit the therapeutic effects of anti-CTLA-4 in head and neck tumors [96].

#### Tumor-extrinsic factors of immunotherapy resistance: host factors

Patient-specific factors, including gender, diet, obesity, and gut microbiota, may impact immunotherapy efficacy [83]. Among these, gut microbiota has garnered significant attention, influencing resistance to ICI therapy. Analysis of the gut microbiome in melanoma patients treated with anti-PD-1 revealed associations between specific bacterial abundances and prolonged progression-free survival [97]. Gut microbes have been identified as potential determinants or biomarkers of immunotherapy response in various cancers [98, 99].

Other factors contributing to resistance include patient-specific differences in immune function and suboptimal dosing or treatment regimens. In essence, immunotherapy resistance involves intricate interactions among tumor cells, immune cells, cytokines, and signaling pathways. A comprehensive understanding of these mechanisms is crucial for developing effective strategies to overcome resistance and enhance clinical outcomes.

## CircRNAs in regulating response to cancer immunotherapy

### The potential of circRNAs as biomarkers for predicting immunotherapy response

Beyond traditional protein biomarkers like PD-L1, TMB, MSI-H, and dMMR [58, 100–102], circRNAs have emerged as innovative biomarkers for tumor immunotherapy, offering potential targets due to their stability in body fluids and specificity across tissues and developmental stages [103]. Recent findings demonstrate that plasma hsa_circ_0000190 levels are inversely correlated with immunotherapy response in advanced lung cancer patients. Notably, hsa_circ_0000190 exhibits promise as a novel biomarker for immunotherapy effectiveness, independent of PD-L1 expression levels [104]. Zhou et al. identified the inaugural circRNA signature (hsa_circ_0006408, hsa_circ_0032116, hsa_circ_0003633, hsa_circ_0066874, and hsa_circ_0006508) capable of predicting survival benefits in advanced melanoma patients undergoing anti-PD-1 immunotherapy [105]. Another study identified has_CDR1 as a potential response biomarker for effectively predicting anti-PD-1 therapy outcomes in patients with cutaneous metastatic melanoma [106]. Additionally, Gao et al. demonstrated that hsa_circ_0066351, through the construction of risk assessment models, is associated with colorectal cancer prognosis and immunotherapy response [107]. These investigations underscore the potential of circRNAs as valuable biomarkers for predicting immunotherapy response, presenting an opportunity to inform treatment decisions and enhance patient outcomes. However, the clinical application of circRNAs as predictive biomarkers for immunotherapeutic response necessitates further exploration.

### Role of circular RNAs in regulating resistance to cancer immunotherapy (Fig. 4)

Immunotherapy resistance can occur through various mechanisms, including alterations in antigen presentation, reduction of immunogenicity, and activation of negative immune checkpoints, etc. The unique closed-loop structure and biological functions of circRNAs lead to their ability to inhibit or promote tumor progression and mediate resistance to cancer therapy [108]. Here, we summarize the mechanisms by which circRNAs are involved in immunotherapy resistance in different tumors (Table 1).

**Fig. 4:**
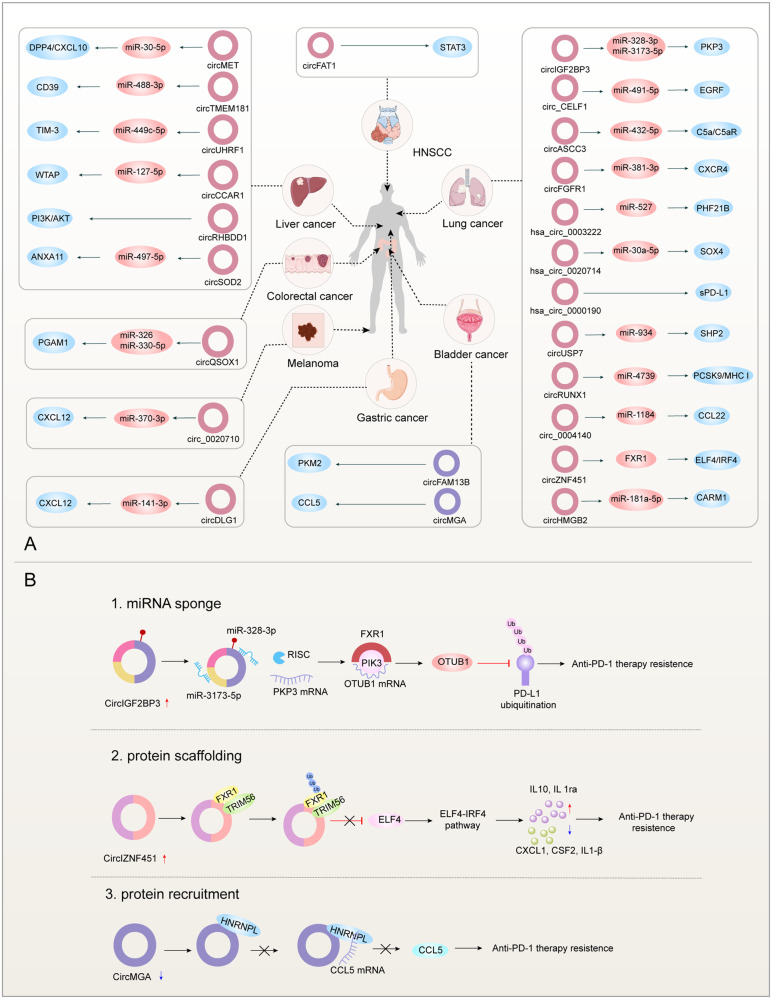
CircRNAs in tumor immunotherapy resistance and molecular mechanisms. **A** CircRNAs in immunotherapy resistance in different tumors. The purple and pink circles represent down-regulated and up-regulated circRNAs, respectively. Orange and blue colors signify downstream target miRNAs and target proteins influenced by circRNAs, respectively. Sections of the figure were sourced from SMART—Servier Medical Art (https://smart.servier.com, last accessed September 17th, 2023). **B** Molecular mechanisms of circRNAs in ICI therapeutic resistance. Illustrated are three exemplar molecular mechanisms featuring CircIGF2BP3, CircIZNF451, and CircMGA contributing to Immune Checkpoint Inhibitor (ICI) therapeutic resistance.

**Table 1:** CircRNAs involved in regulating mechanisms of immunotherapy resistance in various cancers.

Cancer types	CircRNAs	Mechanisms	Roles	Functions	Ref.
Lung cancer	circIGF2BP3↑	miR-328-3p and miR-3173-5p↓→PKP3↑	Reduces CD8+ T cell exhaustion and infiltration	Anti-PD-1 therapy resistance	[110]
	circCELF1↑	miR - 491 - 5p↓→EGRF↑	Restriction of CD8+ T cell recruitment in TME	Anti-PD-1 therapy resistance	[111]
	circASCC3↑	mir-432–5p↓→C5a↑→C5a/C5aR axis ↑	Induces CD8+ T cell depletion, promotes macrophage M2 phenotype, and reshapes immunosuppressive TME	Anti-PD-1 therapy resistance	[112]
	circFGFR1↑	miR-381-3p↓→CXCR4↑	Reduces CD8+ T cell infiltration	Anti-PD-1 therapy resistance	[113]
	hsa_circ_0003222↑	miR-527↓→PHF21B↑	Promotes proliferation and stemness of NSCLC cells	Anti-PD-L1 therapy resistance	[115]
	hsa_circ_0020714↑	miR-30a-5p↓→SOX4↑	Increased expression of the transcription factor SOX4, which acts as an oncogene	Anti-PD-1 therapy resistance	[116]
	hsa_circ_0000190↑	sPD-L1↑	Interferes with T cell activation	Anti-PD-L1 therapy resistance	[114]
	circUSP7↑	miR-934↓→SHP2↑	Inhibits CD8+ T cell function	Anti-PD-1 therapy resistance	[117]
	circRUNX1↑	miR-4739↓→PCSK9↑→MHC I ↓	Reduces CD8+ T cell infiltration and MHC I expression	Anti-PD-1 therapy resistance	[118]
	circ_0004140↑	miR-1184↓→CCL22↑	Recruit Treg cells from TME	Anti-PD-1 therapy resistance	[119]
	circZNF451↑	FXR1↓→ELF4–IRF4 pathway↑	Induces macrophage M2 phenotype and CD8+ T cell exhaustion	Anti-PD-1 therapy resistance	[120]
	circHMGB2↑	miR‑181a‑5p↓→CARM1↑	Inhibit the type 1 INF response	Anti-PD-1 therapy resistance	[121]
Hepatocellular carcinoma	circMET↑	miR-30-5p↓→Snail/ DPP4↑→CXCL10↓	reshapes immunosuppressive TME	Anti-PD-1 therapy resistance	[123]
	circTMEM181↑	miR-488-3p↓→CD38↑→CD39/CD37 axis↑→CD8+ T cell exhaustion	Adenosine accumulation reshapes the immunosuppressive TME	Anti-PD-1 therapy resistance	[124]
	circUHRF1↑	miR-449c-5p↓→TIM-3↑→IFN – γand TNF – α↓	Upregulates inhibitory receptor expression and reduces immune cell infiltration	Anti-PD-1 therapy resistance	[125]
	circCCAR1↑	miR‑127‑5p↓→WTAP↑	Promotes CD8+ T cell exhaustion	Anti-PD-1 therapy resistance	[126]
	circRHBDD1↑	PIK3R1↑→PI3K/AKT pathway↑	Promotes glycolysis and reshapes immunosuppressive TME	Anti-PD-1 therapy resistance	[127]
	circSOD2↑	miR-497-5p↓→ANXA11↑	Inhibition of CD8+ T cell viability	Anti-PD-1 therapy resistance	[128]
Colorectal cancer	circQSOX1↑	miR-326 and miR-330-5p↓→PGAM1↑	Lactate accumulation and increased Treg cells cause immunosuppressive TME	Anti-CTLA-4 therapy resistance	[131]
Bladder cancer	circFAM13B↓	PKM2↑	Increased glycolysis leads to an immunosuppressive TME	Anti-PD-1 therapy resistance	[133]
	circMGA↓	CCL5↓	Decreased infiltration of CD8+ T cells	Anti-PD-1 therapy resistance	[134]
Melanoma	circ_0020710↑	miR-370-3p↓→CXCL12↑	Decreased infiltration of CD8+ T cells and increased infiltration of immunosuppressive cells	Anti-PD-1 therapy resistance	[137]
HNSCC	circFAT1↑	STAT3↑ → CD8+T cells infiltration↓	Enhance cancer cell stemness and reduce CD8+ T cell infiltration	Anti-PD-1 therapy resistance	[140]
Gastric cancer	circDLG1↑	miR-141-3p↓→CXCL12↓	Decreased infiltration of CD8+ T cells and increased infiltration of immunosuppressive MDSCs in TME	Anti-PD-1 therapy resistance	[141]

#### Lung cancer (LC)

Lung cancer is a highly lethal tumor globally. Immunotherapy has improved long-term survival, about 40–50% of patients show resistance in the first cycle [109]. CircRNAs play a pivotal role in diminishing immunotherapy sensitivity through intricate molecular mechanisms across various lung cancer types. Notable examples include CircIGF2BP3 in NSCLC, associated with poor prognosis by reducing CD8+ T cell immune infiltration and upregulating PKP3 expression via sponging miR-328-3p and miR-3173-5p, ultimately preventing PD-L1 degradation and promoting immune escape [110]. CircCELF1 restricts T cell recruitment at the TME and inhibits anti-PD-1 therapy by sponging miR-491-5p, leading to upregulated EGRF expression [111]. CircASCC3 induces C5a activation, remodels the immunosuppressive TME, and induces anti-PD-1 therapeutic resistance [112]. Similarly, CircFGFR1 exerts immunosuppressive effects by sponging miR-381-3p, reducing CD8+ T cell infiltration [113], while hsa_circ_0003222, hsa_circ_0020714 and hsa_circ_0000190 promote proliferation, stemness, and anti-PD-1 therapeutic resistance in NSCLC [114–116]. Exosomal CircUSP7 inhibits CD8+ T cell function, contributing to immunotherapy resistance [117]. In lung adenocarcinoma (LUAD), circRUNX1 affects antigen presentation by sponging miR-4739 [118], and circ_0004140 reduces anti-PD-1 therapeutic effect by promoting CCL22 expression through sponging miR-1184, recruiting Treg cells [119]. Exosomal circZNF451 plays a crucial role in restructuring the tumor-immune microenvironment by influencing macrophage polarization through the FXR1-ELF4–IRF4 axis, serving as a promising biomarker to predict the responsiveness of PD-1 blockade in LUAD [120]. Additionally, circHMGB2 limits PD-1 blockade efficacy by upregulating CARM1 through interacting with miR-181a-5p, inhibiting the type 1 IFN response and desensitizing tumor cells to cytotoxic T-cell-mediated immune responses [121].

#### Hepatocellular carcinoma (HCC)

Liver cancer, a prevalent global malignancy, is predominantly represented by HCC, constituting around 90% of all liver cancer cases. Detection of HCC typically occurs in advanced stages, and despite the incremental survival benefit offered by current therapies, the challenge of immunotherapy resistance persists due to the immunosuppressive TME [122]. Elevated expression of circMET in HCC cells correlates with increased invasion, metastasis, and resistance to immunotherapy. Mechanistically, circMET upregulates snail and its downstream DPP4 by sponging miR-30-5p, fostering immunosuppression through the miR-30-5p/snail/DPP4 axis, leading to CXCL10 degradation. The DPP4 inhibitor Sitagliptin enhances the effectiveness of anti-PD-1 therapy [123]. In HCC, resistance to anti-PD-1 treatment can be induced through tumor-macrophage interactions. Exosome circTMEM181 in the TME upregulates CD39 expression in macrophages, activating the ATP-adenosine pathway and inducing immunosuppression. Targeting the adenosine pathway can enhance the efficacy of ICB therapy [124]. Similarly, exosome-delivered circUHRF1 induces immunosuppressive effects by upregulating TIM-3 expression in NK cells, leading to NK cell exhaustion and inhibiting IFN-γ and TNF-α secretion, ultimately mediating HCC resistance to anti-PD-1 therapy. The underlying molecular mechanism involves circUHRF1-mediated degradation of miR-449c-5p, leading to heightened TIM-3 expression in NK cells and subsequent immunosuppression [125]. Exosome-derived circCCAR1 targets miR-127-5p to upregulate WTAP in activated T cells, causing CD8+ T cell dysfunction and promoting HCC resistance to anti-PD-1 therapy [126]. High levels of CircRHBDD1 are associated with poor patient prognosis and reduced sensitivity to anti-PD-1 therapy, and its inhibition enhances the therapeutic effect of anti-PD-1 by interacting with YTHDF1 to enhance PIK3R1 translation [127]. CircSOD2, highly expressed in HCC tissues, is linked to decreased CD8+ T cell activity in TME, inducing anti-PD-1 therapeutic resistance by binding to miR-497-5p to upregulate ANXA11 [128].

#### Colorectal cancer (CRC)

CRC holds the third position in global cancer incidence and ranks second in cancer-related deaths [129]. Traditional treatments face challenges due to chemotherapy drug side effects and the unique biological characteristics of tumor cells. Although ICB therapy has introduced a new approach, it proves effective in only ~15% of CRC patients with high microsatellite instability (MSI-H). Furthermore, some initially responsive patients develop acquired resistance [130]. In CRC patients, the upregulation of circQSOX1 contributes to resistance to anti-CTLA-4 treatment. Mechanistically, circQSOX1 sponges miR-326 and miR-330-5p, subsequently upregulating the expression of phosphoglycerate mutase 1 (PGAM1), promoting glycolysis in CRC cells. Additional investigations reveal that the knockdown of circQSOX1 reduces the infiltration of immunosuppressive Treg cells, thereby leading to the development of immunotherapy resistance by remodeling the immunosuppressive TME [131].

#### Bladder cancer(Bca)

ICB therapy has demonstrated significant clinical efficacy in BCa, ranking as the ninth most common tumor globally [132]. Despite these advances, the majority of patients do not respond to immunotherapy. A recent study reveals that the downregulation of circFAM13B expression in BCa correlates with a poorer prognosis and resistance to anti-PD-1 treatment. Mechanistically, circFAM13B inhibits the stability of PKM2 mRNA by interacting with IGF2BP1 via the K homology 3–4 (KH3-4) domains, consequently suppressing glycolysis in which PKM2 is involved. Consequently, the decreased circFAM13B fails to inhibit glycolysis, contributing to an immunosuppressive TME. In HuNOG mouse models bearing BCa tumors, the overexpression of circFAM13B enhances the effectiveness of CD8+ T cells and sensitivity to anti-PD-1 therapy [133]. Similarly, the downregulation of circMGA reduces the therapeutic sensitivity of anti-PD-1. CircMGA forms an RNA-protein complex by binding with HNRNPL, leading to the upregulation of CCL5 and increased infiltration of CD8+ T cells, thereby enhancing the immunotherapeutic effect of anti-PD-1 [134].

#### Melanoma

In the nearly decade since the approval of ipilimumab for metastatic melanoma treatment in 2011, despite the initial effectiveness of ICB therapy in 40–45% of patients, the majority eventually develop acquired drug resistance [135, 136]. A study by Wei et al. indicates a positive correlation between circ_0020710 expression and poor prognosis, with upregulated circ_0020710 reducing sensitivity to anti-PD-1 treatment. Mechanistically, circ_0020710 upregulates CXCL12 expression by sponging miR-370-3p. Elevated levels of CXCL12 contribute to the recruitment of immunosuppressive cells, fostering an immunosuppressive microenvironment and resulting in decreased infiltration of cytotoxic T lymphocytes. This ultimately leads to the development of immunotherapeutic resistance [137].

#### Head and neck squamous cell carcinoma (HNSCC)

Preclinical evidence suggests that HNSCC may elude immune surveillance and induce immunosuppression, resulting in a limited response to anti-PD-1 antibodies in most patients [138]. Cancer stem cells, a small subset of tumor cells crucial for self-renewal and promoting malignant tumor development, also contribute to resistance against radiotherapy and chemotherapy [139]. A recent study has unveiled that circRNA can diminish immunotherapy sensitivity by regulating HNSCC cell stemness. In the investigation conducted by Jia et al., circFAT1 expression was found to be upregulated in head and neck squamous cell carcinoma, correlating with poor prognosis and reduced efficacy of immunotherapy. CircFAT1 binds to STAT3 in the cytoplasm, hindering SHP1-mediated dephosphorylation and activating STAT3. Knocking down circFAT1 attenuates tumor stemness and enhances CD8+ T cell infiltration at tumor sites, thereby boosting the effectiveness of anti-PD-1 therapy [140].

#### Gastric cancer

Gastric cancer poses a significant clinical challenge, and despite the introduction of anti-PD-1 therapy, the majority of patients do not achieve favorable outcomes. In a study by Chen et al., the upregulation of circDLG1 was found to be significantly associated with an aggressive tumor phenotype and poor prognosis in gastric cancer patients undergoing anti-PD-1 therapy. Mechanistically, circDLG1 upregulates the expression of CXCL12 by sponging miR-141-3p. The elevated CXCL12 levels further contribute to increased MDSCs and decreased infiltration of CD8+ T cells in TME. Notably, the knockdown of CXCL12 demonstrates the potential to enhance the sensitivity of gastric cancer to anti-PD treatment, suggesting a critical role for circDLG1 in mediating immunotherapeutic responses [141].

## Future directions and discussion

CircRNAs, characterized by a stable covalent closed-loop structure, exhibit tissue-specific and developmental stage-specific distribution. Over the past decade, extensive research has deepened our understanding of the pivotal role of circRNAs in human diseases and physiological processes [142]. In oncology, circRNAs act as miRNA sponges, protein decoys, or translated proteins, exerting regulatory control over tumor growth. Recent explorations have extended their involvement in chemotherapy, radiation therapy, targeted therapy, and immunotherapy resistance. The current focus of circRNA research in immunotherapy resistance is mainly on lung cancer and HCC, particularly in the context of ICB therapy. Mechanistically, circRNAs modulate immunotherapy resistance by binding to miRNAs or serving as protein scaffolds. However, the potential involvement of circRNAs in immunotherapy resistance through other functions remains an area requiring further investigation.

Recently, immunotherapy has garnered significant attention as a highly effective treatment compared to traditional radiotherapy and chemotherapy. Despite achieving high response rates in clinical treatment, the emergence of drug resistance remains a substantial challenge [53]. Research on circRNAs has sparked a new wave in the field of immunotherapy. As scientists delve into understanding the role of circRNAs in regulating resistance to cancer immunotherapy, the identification of novel circRNAs involved in this process could unveil new targets for therapeutic intervention. With over 100,000 different human circRNAs discovered to date, their dysregulation is linked to tumor development across various cancers such as breast, liver, lung, prostate, neuroblastoma, and stomach cancers [143–147]. This dysregulation may contribute to tumor progression or treatment resistance [148, 149]. However, further studies are needed to determine the functionality of differentially expressed circRNAs in normal and tumor tissues, their role in tumorigenesis, development, regulation of immunotherapy resistance, and their potential as drug targets.

PD-L1 and PD-1 are pivotal immune checkpoint proteins targeted in cancer immunotherapy. They regulate immune responses and are significant therapeutic targets. PD-L1, often upregulated in tumors, enables immune evasion by binding to PD-1 on T cells, leading to T cell dysfunction and apoptosis. ICIs that block the PD-1/PD-L1 axis have transformed cancer treatment by reactivating T cell anti-tumor responses. However, resistance to ICIs remains a challenge. Recent studies have focused on circRNAs’ role in modulating the PD-1/PD-L1 pathway, shedding light on novel mechanisms of immunotherapy resistance. For instance, circRNAs like circUHRF1 have been found to be significantly upregulated in oral squamous cell carcinoma (OSCC), and acts as a sponge for miR-526b-5p, leading to increased PD-L1 expression and immune evasion [150]. Similarly, in OSCC, m6A-circRNAs exhibit distinct modification patterns, providing insights into their roles in cancer progression [151]. These findings underscore the intricate regulatory network involving circRNAs, miRNAs, and immune checkpoint proteins in tumor immunotherapy resistance. Understanding this crosstalk is crucial for developing effective strategies to overcome immunotherapy resistance and improve patient outcomes in cancer treatment.

The low cellular content of circRNAs, coupled with the presence of linear mRNA sharing the same sequence, poses a significant obstacle to accurate circRNA identification and detection. The development of more precise detection methods is essential. Current circRNA detection methods include real-time quantitative polymerase chain reaction, droplet digital PCR, circRNA fluorescence in situ hybridization, high-throughput sequencing technology, nanopore sequencing technology, and emerging methods like Duplex-specific nuclease, and loop-mediated isothermal amplification (LAMP) [152]. These varied analytical methods offer scientists more choices, and emerging techniques promise to provide a more robust tool for the clinical application of circRNA.

**Fig. 5:**
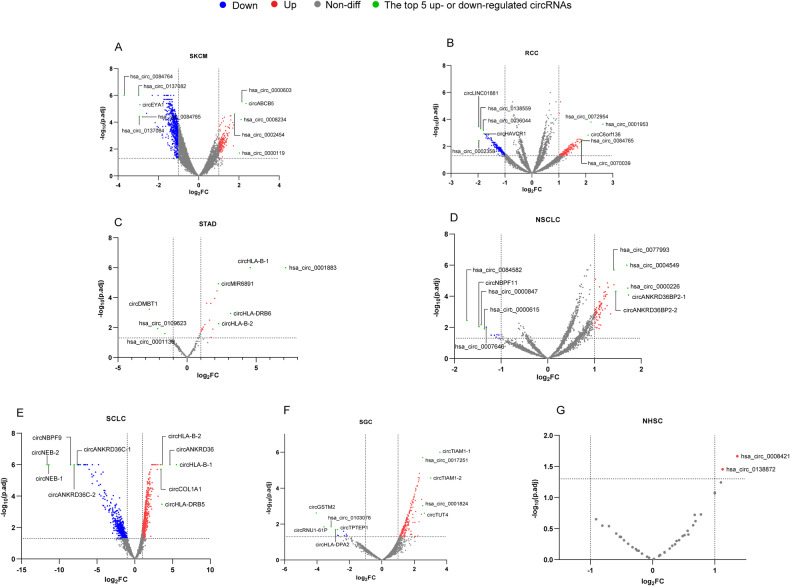
Volcano plots depicting differential expression of circRNAs in immunotherapy responsive and non-responsive patients across seven tumors. **A–G** Differential expression of circRNAs in patients who responded and did not respond to immunotherapy in SKCM, RCC, STAD, NSCLC, SCLC, SGC and NHSC, respectively. The data were sourced from the TCCIA website (http://biotrainee.vip:18888/TCCIA/, last accessed on January 7th, 2024). CircRNAs lacking circBase ID are denoted in the format of circ + parent genes. Abbreviations: SKCM skin cutaneous melanoma, RCC renal cell carcinoma, STAD stomach adenocarcinoma, NSCLC non-small cell lung cancer; SCLC small cell lung cancer, SGC salivary gland cancer, NHSC head and neck squamous cell carcinoma.

The Cancer CircRNA Immunome Atlas (TCCIA), a recently launched website available at http://biotrainee.vip:18888/TCCIA/ or https://shiny.hiplot.cn/TCCIA, stands out as an innovative online platform facilitating the study of circRNA expression and analysis across 25 patient cohorts undergoing immunotherapy targeting CTLA4, PD-1, or PDL-1. This platform serves as a unique avenue to delve into circRNAs, evaluating their potential as biomarkers to predict immunotherapy responses and unraveling their broader implications in cancer research [153]. Volcano plots depicting the differential expression of circRNAs between non-responsive and responsive immunotherapy patients in various tumors were acquired from TCCIA (Fig. 5). The circRNAs, identified with circBase IDs, ranking among the top 5 up-or down-regulated in each tumor were highlighted in green (Log_2_FC ≤ −1 or Log_2_FC ≥ 1; P.adj < 0.05; method, DEseq2). Notably, our analysis revealed a downregulation of several circRNAs transcribed by the EYA1 gene in SKCM. Likewise, in SCLC, multiple circRNAs derived from NEB and ANKRD36C genes exhibited a significant down-regulation trend. We hypothesize that these circRNAs might play a role in tumor immunotherapy resistance or potentially serve as biomarkers for immunotherapy efficacy, and thorough validation through further studies is imperative.

The identification of circRNAs as regulators of immune checkpoint gene expression presents a promising avenue for developing novel therapeutic strategies to enhance the effectiveness of cancer immunotherapy. Techniques such as gene knockout, RNA interference (RNAi), and antisense technologies are currently employed to inhibit the function of oncogenic circRNAs. In a recent study published in Nature Methods, the clustered regularly interspaced short palindromic repeats and associated proteins (CRISPR-Cas)13 system was demonstrated to specifically knock down circRNAs by targeting the back-splice junction (BSJ) instead of intronic complementary sequences [154]. Moreover, targeting signaling pathways influenced by circRNAs, as demonstrated in studies on breast cancer and glioblastoma [155, 156], can also exhibit anti-tumor effects when direct circRNA targeting is challenging.

Additionally, circRNAs can be utilized as tumor antigens or vaccine adjuvants in anti-tumor therapy [157]. Although clinical application of circRNA tumor vaccines is not yet realized, preclinical studies show promising results, with engineered circRNAs expected to be the next generation of RNA vaccines. Currently, three in vivo delivery vectors for circRNAs-Lipid nanoparticles (LNPs), Exosomes, and Virus-like particles (VLPs)-are being explored [158, 159]. LNPs offer advantages such as nuclease resistance, kidney clearance avoidance, and increased cellular uptake. Exosomes, natural endogenous carriers, provide low toxicity, no immunogenicity, and good permeability. VLPs, self-assembled nanocarriers, trigger a strong immune response without containing viral nucleic acids, demonstrating effective tissue targeting and deliverability. Several therapeutic studies have demonstrated the potential of these delivery methods. For example, PEG-Au-loaded circ-Foxo3 expression plasmids enhance the sensitivity of prostate cancer-bearing mice to docetaxel [160]. RGD-modified exosomes carrying circDIDO1 inhibit gastric cancer development [161], and oxaliplatin-resistant cells deliver circRNA ciRS-122 to sensitive cells via exosomes in colorectal cancer, reversing drug resistance in tumor-bearing mice [162]. While these delivery methods hold promise, challenges such as off-target effects, toxicity, standardized manufacturing, quality control, and the need for novel, precise drug delivery systems responsive to enzymes, pH, or hypoxia must be addressed. Comprehensive consideration based on target circRNA, tumor type, and clinical pathology is essential for successful implementation.

The discovery of circRNAs as regulators of immune checkpoint gene expression suggests their potential integration into existing cancer diagnostic and therapeutic workflows, aiming to improve the accuracy and efficacy of cancer treatment and enhance patient outcomes. Early cancer diagnosis remains a significant challenge, emphasizing the need for reliable biomarkers. CircRNAs show promise as biomarkers, capable of differentiating cancer subtypes and guiding treatment decisions. For instance, Tan et al. discovered F-circEA, a novel fusion circRNA specific to EML4-ALK-positive NSCLC patients, serving as a potential diagnostic biomarker and guiding targeted therapy with ALK inhibitors [6, 163]. CircRNAs may aid in immunotherapy selection by detecting their expression in tumor tissues. The expression of PD-L1, considered a biomarker for immunotherapy sensitivity, is intricately regulated by circRNAs, as illustrated in Table 2. However, it is essential to note that elevated PD-L1 expression does not universally guarantee better outcomes in ICB treatment. “Role of circular RNAs in regulating resistance to cancer immunotherapy” highlights the role of circIGF2BP3, which upregulates tumor cell PD-L1 expression, potentially leading to T cell dysfunction-mediated immune escape and immunotherapy resistance. Despite these complexities, circRNAs continue to play a crucial role in tumor diagnosis and treatment.

**Table 2:** CircRNAs regulate the expression of PD-L1 in different cancers.

Cancer types	CircRNAs	Mechanisms	Roles	PD-L1 expression	Ref.
Lung cancer	circRNA-002178↑	miR-34a↓(in tumor cells) ；miR-28-5p↓(in T cells)	May be a biomarker for LUAD diagnosis	PD-L1 ↑ ; PD-1↑	[164]
	circ_0000284↑	miR-377-3p↓	Induces NSCLC cells migration, invasion, and proliferation	PD-L1↑	[165]
	circ-CPA4↑	let-7 miRNA↓	Promotes NSCLC cells growth, stemness and resistance to cisplatin	PD-L1↑	[166]
	circ‑HSP90A↑	miR‑424‑5p↓	Promotes NSCLC cells growth, stemness and inhibits CD8+ T cell function	PD-L1↑	[167]
	hsa_circ_0068252↑	miR-1304-5p↓	Associated with poor prognosis and cisplatin resistance	PD-L1↑	[168]
	circCHST15↑	miR-155-5p and miR-194-5p↓	Associated with clinical staging of tumors and immune evasion	PD-L1↑	[169]
	circ_001678↑	miR-326↓→ZEB1↑	Induces immune escape	Activates PD-1/PD-L1 signal path	[170]
	circ_0014235↑	miR-146b-5p↓→YAP↑	Induces Gefitinib resistance and malignant behaviors	PD-L1↑	[171]
CRC	hsa_circ_0020397↑	miR-138↓→TERT↑	Promotes CRC cell viability and invasion, inhibit apoptosis	PD-L1↑	[172]
	CDR1‑AS↑	CMTM4 and CMTM6↑	Associated with poor prognosis	PD-L1↑	[173]
	hsa_circ_0136666↑	miR-497↓	Accelerates CRC cell proliferation, inhibits apoptosis, and promotes immune escape	PD-L1↑	[174]
HCC	circCORO1C↑	NF- κB↑→c-Myc and COX-2↑	Promotes the proliferation, invasion, and metastasis of HCC cells	PD-L1↑	[175]
	circPRDM4	circPRDM4 binds to HIF-1α and recruits it to the CD274 promoter, promoting HIF-1α-mediated transcriptional activation of PD-L1	Promotes tumor growth and immune escape, and its high expression is associated with better anti-PD-1 mAb treatment	PD-L1↑	[176]
HNSCC	circ_0000052↑	miR-382-3p↓	Promotes migration, invasion, and proliferation of HNSCC cells	PD-L1↑	[177]
nasopharyngeal carcinoma	circBART2.2↑	Bind to RIG-I protein→ activate IRF3 and NF-kB	Cause immune escape of nasopharyngeal carcinoma	PD-L1↑	[178]
breast cancer	hsa_circ_0001598↑	miR‑1184↓	Induces trastuzumab‑resistance	PD-L1↑	[179]
	hsa_circ_0067842↑	Interacts with HuR, affecting its nuclear transposition→ CMTM6↑	CMTM6 not only enhances the migration and invasion ability of BC cells but also affects the ubiquitination of PD-L1 and inhibits its degradation.	PD-L1↑	[180]
Diffuse large B-cell lymphoma	circPCBP2↑	miR-33a/b↓	Induce tumor cell stemness and CHOP resistance	PD-L1↑	[181]

(Note: circBART2.2 is encoded by the Epstein–Barr virus in nasopharyngeal carcinoma).

## Conclusions

In this review, we introduce the biogenesis and biological functions of circRNAs, various immunotherapy methods, and factors linked to tumor immunotherapy resistance. We also explore the potential of circRNAs as biomarkers for predicting immune responses and summarize their current regulatory mechanisms in different tumor immunotherapy resistance scenarios. In current studies on immunotherapeutic resistance, circRNAs predominantly either promote or alleviate drug resistance by serving as miRNA sponges or protein decoys. However, the regulation of immunotherapy resistance by circRNAs might involve other mechanisms beyond these, warranting exploration. Uncovering additional mechanisms of circRNAs in immunotherapy resistance could lead to the identification of new therapeutic targets or biomarkers, enhancing tumor diagnosis and prognosis accuracy, enabling precise personalized treatment, and improving overall patient outcomes. The substantial potential of circRNAs in tumor treatment is promising, but the translation of preclinical research into clinical practice requires careful consideration.
